# Recent malaria does not substantially impact COVID-19 antibody response or rates of symptomatic illness in communities with high malaria and COVID-19 transmission in Mali, West Africa

**DOI:** 10.3389/fimmu.2022.959697

**Published:** 2022-08-03

**Authors:** John Woodford, Issaka Sagara, Halimatou Diawara, Mahamadoun Hamady Assadou, Abdoulaye Katile, Oumar Attaher, Djibrilla Issiaka, Gaoussou Santara, Ibrahim H. Soumbounou, Seydou Traore, Moussa Traore, Oumar M. Dicko, Sidi Mohamed Niambele, Almahamoudou Mahamar, Bourama Kamate, Bayaya Haidara, Kourane Sissoko, Seydou Sankare, Sadio dite Koni Diarra, Amatigue Zeguime, Justin Y. A. Doritchamou, Irfan Zaidi, Alassane Dicko, Patrick E. Duffy

**Affiliations:** ^1^ Laboratory of Malaria Immunology and Vaccinology, National Institute of Allergy and Infectious Diseases, Bethesda, MD, United States; ^2^ Malaria Research and Training Center, University of Sciences, Techniques, and Technologies of Bamako, Bamako, Mali

**Keywords:** COVID-19, malaria, falciparum, serology, severity, West Africa

## Abstract

Malaria has been hypothesized as a factor that may have reduced the severity of the COVID-19 pandemic in sub-Saharan Africa. To evaluate the effect of recent malaria on COVID-19 we assessed a subgroup of individuals participating in a longitudinal cohort COVID-19 serosurvey that were also undergoing intensive malaria monitoring as part of antimalarial vaccine trials during the 2020 transmission season in Mali. These communities experienced a high incidence of primarily asymptomatic or mild COVID-19 during 2020 and 2021. In 1314 individuals, 711 were parasitemic during the 2020 malaria transmission season; 442 were symptomatic with clinical malaria and 269 had asymptomatic infection. Presence of parasitemia was not associated with new COVID-19 seroconversion (29.7% (211/711) vs. 30.0% (181/603), p=0.9038) or with rates of reported symptomatic seroconversion during the malaria transmission season. In the subsequent dry season, prior parasitemia was not associated with new COVID-19 seroconversion (30.2% (133/441) vs. 31.2% (108/346), p=0.7499), with symptomatic seroconversion, or with reversion from seropositive to seronegative (prior parasitemia: 36.2% (64/177) vs. no parasitemia: 30.1% (37/119), p=0.3842). After excluding participants with asymptomatic infection, clinical malaria was also not associated with COVID-19 serostatus or symptomatic seroconversion when compared to participants with no parasitemia during the monitoring period. In communities with intense seasonal malaria and a high incidence of asymptomatic or mild COVID-19, we did not demonstrate a relationship between recent malaria and subsequent response to COVID-19. Lifetime exposure, rather than recent infection, may be responsible for any effect of malaria on COVID-19 severity.

## Introduction

Despite considerable concern following the emergence of COVID-19, parts of sub-Saharan Africa have been spared from much of the predicted health burden. This has led to the hypothesis that malaria exposure, among other exposures and demographic factors, may reduce COVID-19 severity (1, 2). While the concept of malaria-induced tolerance/trained immunity is not new, the nature and degree of cross-tolerance for other infections remains uncertain (3–6). In Kenyan children with influenza, malaria co-infection has been associated with a lower incidence of acute respiratory distress syndrome (ARDS), and in a mouse model of malaria and pneumovirus infection, coinfection has been associated with reduced pulmonary inflammation (7, 8). Although nation-level statistics may suggest an association between malaria and COVID-19, community and individual-level data and hypothesis-testing experimental studies are sparse (9). Most case series reporting co-infection are small with high heterogeneity and limited control information, making summary datasets challenging to interpret (10). In a large study of adults hospitalized with COVID-19 in Uganda, prior malaria exposure was associated with less severe and critical COVID-19, and acute *Plasmodium falciparum* co-infection was not found to be deleterious on the clinical course of COVID-19 (11). In contrast, in a study of hospitalized Sudanese adults, acute co-infection was associated with increased mortality (12). As a result, it remains unclear how and if malaria affects COVID-19, particularly in the community where most infections occur.

While observational co-infection studies are critical to describe interactions between COVID-19 and malaria, these studies are challenging to conduct and may be rendered even more difficult by pandemic-related disruptions in already resource-limited settings. We are currently conducting a longitudinal cohort serosurvey to understand the cumulative incidence and clinical burden of COVID-19 in Mali, West Africa, and have found evidence of substantial virus transmission in several communities and seasonal variations in background symptom reporting rates over the course of the 2020 malaria season (13). While there have been some concerns regarding the performance of COVID-19 serology in malaria-endemic regions (14, 15), the two-antigen test used in this study has previously been qualified for use in the target population, with particular attention to understanding and mitigating background reactivity (15, 16). Having confirmed viral transmission in our study communities, we wished to evaluate if seasonal malaria affected responses to COVID-19 in the population. Malaria is highly seasonal in Mali, with intense transmission during the wet season followed by limited transmission during the dry season (17, 18). We present a subgroup analysis of serosurvey participants who also underwent malaria/parasitological surveillance during the 2020 malaria transmission season. In this analysis, we sought to broadly examine if participants with parasitemia during the 2020 malaria transmission season experienced different COVID-19 seroconversion rates, symptom reporting rates, or altered rates of COVID-19 antibody signal change over time compared to participants without parasitemia.

## Materials and methods

Malian individuals participating in the longitudinal cohort COVID-19 serosurvey who also underwent intensive malaria surveillance in clinical trials of investigational malaria vaccines during the 2020 transmission season were included in this subgroup analysis. This cohort was used to assess the rate of new COVID-19 seroconversion and rate of reported symptoms with respect to confirmed parasitemia during the 2020 malaria transmission season. The effect of recent prior parasitemia was also assessed by examining the incidence of these events over the subsequent dry season. Parasitemia episodes were stratified into clinical malaria and asymptomatic malaria, and study participants were stratified into adults (aged 18 years and older) and children. Further analysis was performed by looking at the rates of change in antigen reactivity between study visits based on parasitemia status. This was to help understand if parasitemia affected the specificity or durability in antibody responses (14).

### COVID-19 longitudinal cohort serosurvey

**Figure 1:**
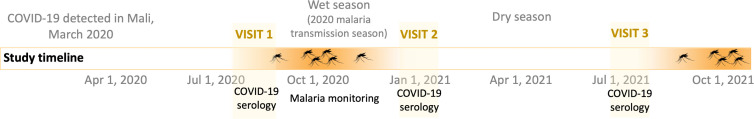
Study overview.

The COVID-19 serosurvey is an ongoing study adapted from the WHO early investigation protocols as previously described (13). In brief, residents of participating Malian communities were invited to provide blood samples for SARS-CoV-2 serology and report any history of symptoms since the onset of the pandemic at study visit 1 in July-August 2020, approximately coinciding with the onset of the malaria transmission season. Participants were subsequently invited to attend for follow up at study visit 2 at around the end of the malaria transmission season in December 2020-January 2021 for repeat SARS-CoV-2 serology and to report symptoms history since the previous study visit. A further follow up visit was conducted in July 2021 at study visit 3 (**Figure 1**). COVID-19 serostatus was assessed using a two-antigen ELISA as previously described (16). In brief, a 1:400 dilution of participant plasma was tested for antibodies to SARS-CoV-2 spike and receptor binding domain (RBD) antigens separately using a semi-automated ELISA developed at the NIH (19). The assay was adapted for use in Mali by establishing population specific cutoffs for each antigen by ROC analysis of local positive and negative control samples (16). A positive result was defined as a signal above cutoff for both spike and RBD. Participants with a positive result that had a negative result at an earlier visit were considered new seroconversions. Symptoms history was collected by asking participants to recall any of a fixed list of 15 symptoms during the prior reporting period (fever, chills, myalgia, fatigue, headache, cough, rhinorrhea, sore throat, shortness of breath, wheeze, anosmia/loss of taste, other respiratory, nausea/vomiting, diarrhea, and abdominal pain) and estimating the overall symptom duration in days. Parental consent and assistance with clinical history was sought for participating children.

### Malaria monitoring

Individuals included in this substudy underwent intensive parasitological monitoring for *P. falciparum* infection during the 2020 wet season (malaria transmission season) as a part of clinical trials at study sites in Doneguebougou (NCT03917654 (20)), Bancoumana (NCT03952650 (21)), and Ouelessebougou (NCT03989102 (22)). In these trials, antimalarial treatment was administered at the beginning of the study to clear any existing parasitemia. Monitoring consisted of frequent scheduled and unscheduled assessments. Scheduled assessments included blood collection for smear microscopy. Unscheduled assessments included *P. falciparum* specific HRP2 rapid detection tests at the discretion of study staff, followed by blood sample collection for gold-standard smear microscopy if positive. Parasitemia was defined as any positive diagnostic test and then stratified into clinical malaria or asymptomatic malaria. Clinical malaria was determined by study clinicians in keeping with local criteria for the syndrome (a positive test accompanied by suggestive symptoms including non-specific symptoms such as fever, headache, and myalgia). Clinical malaria episodes between COVID-19 serosurvey visits 1 and 2 were extracted from the MEDDra coded adverse event database of each clinical trial and matched to individual serosurvey participants. Asymptomatic infections identified between COVID-19 serosurvey visits 1 and 2 was extracted from the laboratory results database of each clinical trial and matched to individual serosurvey participants. Asymptomatic malaria was defined as a positive diagnostic test in the absence of a clinical malaria adverse event. All malaria diagnoses were managed per Malian guidelines.

### Analysis

Data analysis was performed across two time periods. The first period was between serosurvey visit 1 and 2, approximately coinciding with the 2020 malaria transmission season and intensive malaria monitoring. This period was used to examine the effect of parasitemia on COVID-19 seroconversion and symptom reporting rates. The second period was between serosurvey visit 2 and 3, coinciding with the dry season. This period was used to assess the effect of recent prior parasitemia on subsequent new COVID-19 seroconversion and symptom reporting rates. Participants with parasitemia were stratified into clinical malaria and asymptomatic malaria groups. Any history of clinical malaria was sufficient for stratification into the clinical malaria group. Reported symptoms in individuals with COVID-19 seroconversion alone versus COVID-19 seroconversion plus parasitemia were compared to assess the effect of parasitemia on symptomatic COVID-19. In addition to categorical seroconversion rates, the rate of change in COVID-19 antigen reactivity between study visits was assessed stratified by the presence of parasitemia during the 2020 malaria transmissions season to understand if parasitemia affected longitudinal COVID-19 antibody response. Finally, rates of seropositivity were compared between participants with confirmed parasitemia within 30 days prior to sample collection and those with more distant parasitemia to understand if recent parasitemia affected serostatus.

Demographic data, parasitemia rates, and COVID-19 seroconversion rates were presented descriptively. Continuous variables were presented as mean and 95% confidence interval (CI) or median and interquartile range (IQR) depending on the normality of distribution. A Fisher exact test was used to compare groups for categorical variables, and a student t-test or Mann Whitney U test used to compare groups for continuous variables depending on the normality of distribution. To understand the effect of parasitemia on new COVID-19 seroconversion, a logistic regression was performed that included parasitemia, site, age, and sex as the independent variables. Demographic factors have previously been associated with seropositivity in Mali (13). The effect of parasitemia on reported symptoms was assessed by comparing individuals with seroconversion alone and individuals with seroconversion and recent parasitemia. The Holm-Sidak method was used to correct for multiple comparisons when assessing groups of reported symptoms. To help understand if the reported symptoms collected in the COVID-19 serosurvey were meaningful, a logistic regression was performed using confirmed clinical malaria as the dependent variable to identify if any symptoms were independently associated with clinician-confirmed symptomatic clinical malaria. Standardized rates of change in COVID-19 antigen reactivity between study visits were presented by calculating the rate of assay signal change for spike and RBD antigens per 100 days in participants stratified by parasitemia and COVID-19 serostatus.

An age-stratified analysis of adults (18 years and older) and children (under 18 years) was also performed to describe COVID-19 seroconversion, symptom reporting, and the changes COVID-19 antigen reactivity over time based on the presence of parasitemia during the 2020 malaria transmission season.

Statistical analyses were performed using GraphPad Prism 9.

## Results

There were 1321 individuals who completed serosurvey visit 1 and visit 2 and underwent malaria monitoring over the 2020 transmission season. Of these 1321 individuals, seven were COVID-19 seropositive at visit 1 and were removed from the analysis as the time of COVID-19 seroconversion did not occur during the study period. The cohort had a median age of 20 years (IQR 9 to 37), was 49.5% male (651/1314), and had very few medical comorbidities (0.9% (12/1314) (**Table 1**). Of the 1314 eligible individuals, 1092 completed serosurvey visit 3 after the dry season. Due to budget restrictions participants at the Bancoumana site did not undergo visit 3 follow up. Nine participants were removed from the visit 3 analysis due to intercurrent COVID-19 vaccination as serostatus due to natural infection could no longer be assessed. No other participants reported COVID-19 vaccination during the study period. Participants underwent a median 10 (IQR 6-12) diagnostic tests for malaria monitoring during the study period, corresponding approximately to second or third weekly testing during the 2020 malaria transmission season. Clinical malaria and asymptomatic malaria episodes were recorded at all sites.

**Table 1:** Study population (n=1314) and participants stratified by parasitemia status during 2020 malaria transmission season.

	Overall population (n=1314)	No parasitemia during 2020 season (n=603)	Parasitemia during 2020 season (n=711)	P value
Age, years (median, IQR)	20 (9-37)	27 (9-40)	16 (10-32)	<0.0001
Sex, male	49.5% (651/1314)	46.1% (278/603)	52.5% (373/711)	0.0232
Comorbidity	0.9% (12/1314)	1.2% (7/603)	0.7% (5/711)	0.4012
New visit 2 seroconversion	29.8% (392/1314)	30.0% (181/603)	29.7% (211/711)	0.9038
New visit 3 seroconversion	30.6% (241/787)	31.2% (108/346)	30.2% (133/441)	0.7499
Visit 2 seropositives reverting to seronegative by visit 3	34.1% (101/296)	30.1% (37/119)	36.2% (64/177)	0.3842

Comorbidity refers to any of the following self-reported chronic conditions at the time of enrolment: obesity, diabetes, HIV or other immunosuppression, hypertension, cardiovascular disease, chronic lung disease, chronic hematological disorder, chronic kidney disease, chronic neurological impairment, malignancy.

### Visit 1 to visit 2 (2020 malaria transmission season; wet season)

Between visit 1 and visit 2, 29.8% (392/1314) of participants demonstrated new COVID-19 seroconversion and 54.1% (711/1314) had at least one positive malaria diagnostic test. Among these, 442 participants had at least one episode of clinical malaria and 269 had asymptomatic malaria only. Smear data were available for 99.3% (706/711) of episodes, and 99.7% (709/711) of tests detected *P. falciparum.* There were two non-*P. falciparum* mono-infections; one *P. ovale* and one *P. malariae*.

Overall, participants with parasitemia during the 2020 malaria transmission season were younger and more likely to be male compared to those with no parasitemia (median age 16 years IQR 10 to 32 vs. 27 years IQR 9 to 40, p<0.0001; 52.5% male (373/711) vs. 46.1% male (278/603), p=0.0232) but otherwise similar with respect to demography and comorbidity profiles (**Table 1**). After stratifying into adults and children, the difference in sexes was only apparent in children, where parasitemia during the transmission season was more common in older, male children (**Supplementary Table 1**). Among participants with confirmed parasitemia, those with clinical malaria had higher parasite counts at diagnosis and were older compared to asymptomatic malaria, primarily driven by results in children (median parasitemia 158 p/μL IQR 17 to 900 *vs*. 9 p/μL IQR 3 to 118, p<0.0001, median age 18 years IQR 11 to 33 vs. 14 years IQR 5 to 30, p=0.0011; **Supplementary Tables 2**, **3**). There were 211 participants with new COVID-19 seroconversion and parasitemia (median age 24 years IQR 14 to 35, 49.8% (105/211) male). Of these, 141 had clinical malaria.

Parasitemia during the 2020 malaria transmission season did not affect COVID-19 seroconversion rates at visit 2 (parasitemia: 29.7% seroconverted (211/711) vs. no parasitemia: 30.0% seroconverted (181/603), p=0.9038; **Table 1**). While there was a slightly lower rate of new seroconversions in participants with asymptomatic malaria compared to those with clinical malaria (asymptomatic malaria: 26.0% (70/269) vs. 31.9% (141/442), p=0.5418, **Supplementary Table 2**), this did not reach statistical significance. In participants with multiple episodes of clinical malaria during the monitoring period there was no difference in COVID-19 seroconversion rates based on the number of episodes (single episode: 33.6% seroconverted (116/345) vs. >1 episode 25.8% seroconverted (25/97), p=0.1748).

Overall, participants with parasitemia during the 2020 malaria transmission season had a greater increase in spike reactivity over time between study visit 1 and 2 compared to those without parasitemia, particularly in children and primarily driven by small absolute differences in seronegative participants (**Supplementary Figures 1**, **2**). No difference was observed in the rate of change in RBD reactivity.

**Figure 2:**
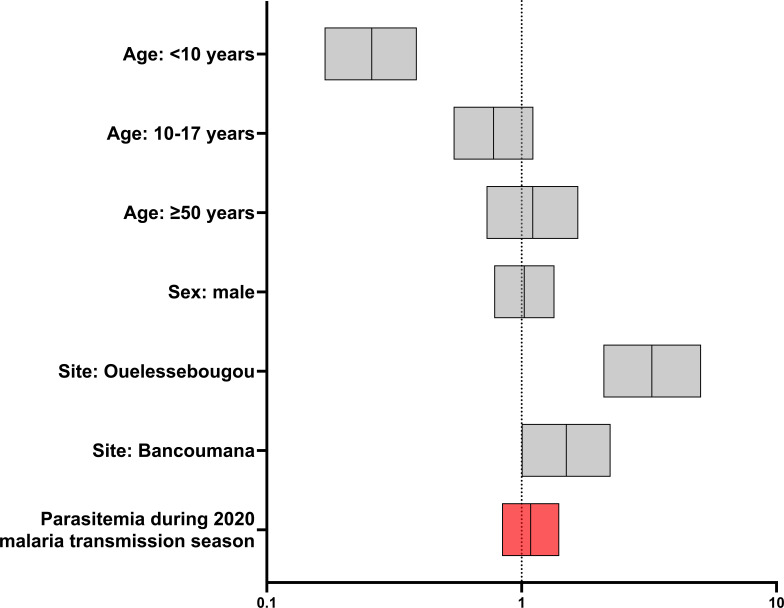
Effect of parasitemia and selected demographic covariates on new COVID-19 seroconversion during 2020 malaria transmission season (n=1314). Forest plot of odds ratios and 95% confidence intervals following logistic regression. Age group 18-49 years and Doneguebougou site were used as reference groups for nominal variables.

In a logistic regression accounting for demographic factors (age group, sex, site), the presence of parasitemia during the 2020 malaria transmission season was not independently associated with COVID-19 seropositivity (OR 1.085 95% CI: 0.8383 to 1.406; **Figure 2**). As expected, age group and site were associated with serostatus. When parasitemia was stratified into clinical malaria and asymptomatic malaria, neither was independently associated with seropositivity following logistic regression (clinical malaria: OR 1.061 95% CI: 0.7955 to 1.414, asymptomatic malaria: OR 1.131 95% CI 0.7965 to 1.599.)

To understand the possible impact of parasitemia during the 2020 malaria transmission season on the clinical presentation of new COVID-19 seroconversion, reported symptoms were compared between participants with new COVID-19 seroconversion plus parasitemia, and new seroconversion alone. The proportion of asymptomatic seroconversions was the same between these groups (parasitemia: 43.1% (91/211) vs. no parasitemia: 43.1% (78/181), p>0.9999). In participants reporting symptoms during the seroconversion period, the presence of parasitemia was not associated with the number of individual symptoms reported or the reported duration of symptoms (parasitemia: median number of symptoms 2 (IQR 1 to 2) vs. no parasitemia: 2 symptoms (IQR 1 to 3), p=0.6364; parasitemia: median duration 3.5 days (IQR 2 to 5) vs. no parasitemia: 4 days (IQR 2 to 5), p=0.3820). There was no difference in the reporting rate of any individual symptom (**Supplementary Table 4**). When stratified by age, children had overall higher rates of symptom reporting, and adult participants with COVID-19 seroconversion and parasitemia reported fewer respiratory symptoms, mainly driven by a lower incidence of rhinorrhea, although this did not reach statistical significance (parasitemia: 31.8% (41/129) vs. no parasitemia: 40.0% (60/150), **Supplementary Table 5**).

To help understand the accuracy of symptom history, self-reported symptoms were assessed in participants stratified by clinician-confirmed clinical malaria. In a logistic regression using clinical malaria as the dependent variable, age group 10-17 years (OR 2.58 95% CI: 1.86 to 3.60), self-reported history of fever (OR 1.79 95% CI: 1.08 to 2.96), and self-reported history of headache (OR 1.44 95% CI 1.07 to 1.94) were independently associated with clinical malaria during the reporting period (**Supplementary Figure 3**).

To help understand if more recent parasitemia affected COVID-19 seropositivity rates, participants with positive malaria diagnostic testing were stratified based on time between confirmed parasitemia and sample collection for COVID-19 serology. Time since parasitemia was not associated with COVID-19 serostatus at visit 2 (≤30 days: 26.0% seroconverted (59/227) vs. >30 days: 31.4% seroconverted (152/484), p=0.1589).

### Visit 2 to visit 3 (dry season)

Between visit 2 and visit 3, 241 participants demonstrated new COVID-19 seroconversion. There were 296 seropositive participants at visit 2 that could be followed to assess signal waning and the rate of reversion to seronegative.

Prior parasitemia during the 2020 malaria transmission season did not affect subsequent new COVID-19 seroconversion rates (prior parasitemia: 30.2% (133/441) *vs*. no parasitemia: 31.2% (108/346), p=0.7499; **Table 1**). Among the 296 participants seropositive at visit 2, recent prior parasitemia did not affect the proportion of participants that reverted to seronegative at visit 3 (prior parasitemia: 36.2% (64/177) vs. no parasitemia: 30.1% (37/119), p=0.3842, **Table 1**). In participants with prior parasitemia, 40.2% (43/107) with clinical malaria and 30.0% (21/70) with asymptomatic malaria seroreverted by visit 3 (p=0.2013, **Supplementary Table 2**). After stratifying by age, this was most pronounced in adults (**Supplementary Table 3**). The rate of signal change between visit 2 and 3 for spike and RBD was not statistically different between groups stratified by recent prior parasitemia or by age (**Supplementary Figures 4**, **5**).

The proportion of asymptomatic new COVID-19 seroconversions was also the same regardless of prior parasitemia status (prior parasitemia: 74.4% (99/133) vs. no parasitemia: 67.6% (73/108), p=0.2549; **Supplementary Table 6**). The number of individual symptoms reported in symptomatic participants and overall reported duration were also the same (prior parasitemia: median number of symptoms 2 (IQR 1 to 2) vs. no parasitemia: 2 (IQR 1 to 2), p=0.4924; prior parasitemia: median duration 3 days (IQR 2 to 4) vs. no parasitemia: 3 days (IQR 2 to 5), p=0.0823). There was no difference in the reporting rate of any individual symptom (**Supplementary Table 6**). Over this time period, symptom reporting rates were lower overall in adults and children compared to rates over the wet season (**Supplementary Table 7**).

## Discussion

In this analysis of individuals participating in a longitudinal cohort COVID-19 serosurvey in Malian communities that experience intense seasonal malaria transmission, we did not identify a substantial effect of asymptomatic malaria or clinical malaria on COVID-19 seroconversion or on the symptoms reported with COVID-19 seroconversion.

In our study population, malaria and COVID-19 seroconversion affected different demographic groups. Positive malaria diagnostic testing was more common in younger participants, with clinical malaria more common in the 10-17 year old group compared to adults, which may be the result of recurrent exposures or seasonal malaria chemoprevention in the participating communities. Conversely, COVID-19 seroconversions were associated with older age which has been previously reported in an interim assessment of our serosurvey (13).

Despite a relatively large sample size and a high incidence of COVID-19 seroconversions and positive malaria diagnostic tests, a substantial interaction between these diseases was not apparent. While we did not demonstrate a relationship between malaria and COVID-19, this does not fully exclude meaningful interactions between the two. Firstly, the high COVID-19 seroconversion rates in our study communities suggest that malaria may not substantially reduce virus transmission, perhaps consistent with experimental murine pneumovirus studies where malaria co-infection did not affect viral shedding (8). Secondly, although recent parasitemia was not associated with COVID-19 serostatus or reported symptoms, it is possible that lifetime cumulative malaria exposure could affect COVID-19 severity rather than the recent exposures assessed in this study. While *P. falciparum* infection of malaria-naïve adults and children may result in a short-lived response (23, 24), a sustained skewing of monocytes towards a regulatory phenotype appears to require multiple exposures over the course of several years (4). As a result, malaria-induced trained immunity or cross tolerance sufficient to affect COVID-19 may require incremental and cumulative malaria exposure. Furthermore, other exposures in sub-Saharan Africa could potentially modulate COVID-19 severity, including BCG vaccination (25) and helminth infection (26). In our study population, BCG vaccine coverage was high (13) and the prevalence of helminth carriage in the study communities has been historically modest (27). It will be interesting in future studies to assess age and serology stratified rates of symptomatic illness in the community and assess any relationship with serological markers of cumulative malaria exposure.

To our knowledge this is the largest longitudinal cohort study combining COVID-19 and malaria surveillance to date, and one of the only studies assessing a community rather than hospitalized population. Nonetheless, there are several limitations. While gold-standard diagnostics were used for malaria, COVID-19 detection was performed using paired serological testing. Although assay performance has been optimized for use in Mali and we did not find evidence of malaria-induced cross-reactivity, the nature of paired testing for seroconversion means only a time window for COVID-19 exposure is available. In Mali, access to PCR diagnostics is limited, and primarily used for hospitalized patients and travelers. Additionally, although microscopy is the gold-standard for malaria diagnosis, it may miss very low parasitemia infections. In this study, reported symptoms cannot be exclusively attributed to a particular event. Despite this, we would expect background reporting rates to be similar across groups to allow for meaningful comparisons, and the independent association of reported fever and headache in the cohort with clinical malaria helps confirm the utility of the symptoms history that was collected. Additionally, although malaria is highly seasonal in our study communities, there is transmission outside of this time, and there is the possibility of parasitemia in participants during the dry season. Finally, we were unable to assess severe complication rates due to the rarity of such events in our study population.

Malaria-induced COVID-19 tolerance remains an interesting hypothesis to explain the relative sparing of sub-Saharan Africa from pandemic-related morbidity. Based on our observations, recent malaria seems unlikely to substantially reduce viral transmission or affect COVID-19 illness rates in the community. It is possible that any tolerance may be related to cumulative malaria exposure rather than concurrent or recent parasitemia.

## Data availability statement

The raw data supporting the conclusions of this article will be made available by the authors, without undue reservation.

## Ethics statement

The COVID-19 serosurvey was conducted as a public health surveillance activity in collaboration with the Malian Ministry of Health and was approved by the Malian ethics committee of Facultes de Medicine/d’Odonto-Stomatologie et de Pharmacie (2020/114/CE/FMOS/FAPH) and the Malian COVID-19 Scientific Review Committee. The malaria studies were approved by the Malian and NIH ethics committees. Written informed consent or assent was obtained from all participants.

## Author contributions

JW: conceptualization, data curation, formal analysis, writing (original draft and review/editing); IS, AD, and PD: conceptualization, supervision, writing (review/editing), funding acquisition; HD, MA, AK, OA, and AZ: data curation, investigation, resources; DI, GS, IHS, ST, MT, OD, SN, AM. BK, BH, KS, SS, and SK: investigation; IZ and JD: validation, data curation, methodology. All authors contributed to the article and approved the submitted version.

## Funding

This project was funded by the Intramural Research Program of the National Institute of Allergy and Infectious Diseases, National Institutes of Health. Clinical trial NCT03917654 was conducted as part of the EDCTP2 program supported by the European Union (grant number RIA2018SV-2311: 2019-2024).

## Conflict of interest

The authors declare that the research was conducted in the absence of any commercial or financial relationships that could be construed as a potential conflict of interest.

## Publisher’s note

All claims expressed in this article are solely those of the authors and do not necessarily represent those of their affiliated organizations, or those of the publisher, the editors and the reviewers. Any product that may be evaluated in this article, or claim that may be made by its manufacturer, is not guaranteed or endorsed by the publisher.
