# Supplementary material for: Recent malaria does not substantially impact COVID-19 antibody response or rates of symptomatic illness in communities with high malaria and COVID-19 transmission in Mali, West Africa
Source: Front Immunol. 2022 Aug 3;13:959697. doi: 10.3389/fimmu.2022.959697 (PMC9382593; doi:10.3389/fimmu.2022.959697)

Supplementary Table 1: Study population stratified into adult participants (aged ≥18 years) and child participants (aged <18 years) and further stratified by parasitemia status during 2020 malaria transmission season

	Adults (n=701)	Adults			Children (n=613)	Children		
		No parasitemia during the 2020 malaria transmission season (n=375)	Parasitemia during the 2020 malaria transmission season (n=326)	P value		No parasitemia during the 2020 malaria transmission season (n=228)	Parasitemia during the 2020 malaria transmission season (n=385)	P value
Age, years (median, IQR)	35 (27-46)	37 (29-48)	33 (25-45)	0.0032	9 (5-12)	7 (4-11)	10 (6-13)	<0.0001
Sex, male	42.9% (301/701)	43.7% (164/375)	42.0% (137/326)	0.7022	57.1% (350/613)	50.0% (114/228)	61.3% (236/385)	0.0069
Comorbidity	1.7% (12/701)	1.6% (6/375)	1.5% (5/326)	>0.9999	0.2% (1/613)	0% (0/228)	0.3% (1/385)	>0.9999
New visit 2 seroconversion	39.8% (279/701)	40.0% (150/375)	39.6% (129/326)	0.9384	18.4% (113/613)	13.6% (31/228)	21.3% (82/385)	0.0179
New visit 3 seroconversion	38.6% (117/303)	39.6% (61/154)	37.6% (56/149)	0.7250	25.6% (124/484)	24.5% (47/192)	26.4% (77/292)	0.6713
Visit 2 seropositives reverting to seronegative by visit 3	34.6% (65/188)	31.1% (28/90)	37.8% (37/98)	0.3605	33.3% (36/108)	31.0% (9/29)	34.2% (27/79)	0.8213

Comorbidity refers to any of the following self-reported chronic conditions at the time of enrolment: obesity, diabetes, HIV or other immunosuppression, hypertension, cardiovascular disease, chronic lung disease, chronic hematological disorder, chronic kidney disease, chronic neurological impairment, malignancy.

P values denote pairwise comparison between no parasitemia and parasitemia groups.

Supplementary Table 2: Participants with positive malaria diagnostic test during 2020 malaria transmission season stratified into clinical malaria and asymptomatic malaria groups

	Any parasitemia during the 2020 malaria transmission season (n=711)	Clinical malaria (n=442)	Asymptomatic malaria (n=269)	P value
Age, years (median, IQR)	16 (10-32)	18 (11-33)	14 (5-30)	0.0011
<5 years	7.0% (50/711)	0% (0/442)	18.6% (50/269)	<0.0001
5-9 years	17.7% (126/711)	17.2% (76/442)	18.6% (50/269)	0.6856
10-17 years	29.4% (209/711)	32.1% (142/442)	24.9% (67/269)	0.0420
18-49 years	40.0% (270/711)	41.2% (182/442)	32.7% (88/269)	0.0258
≥50 years	7.9% (56/711)	9.5% (42/442)	5.2% (14/269)	0.0442
Sex, male	52.5% (373/711)	51.4% (227/442)	54.3% (146/269)	0.4860
Comorbidity	0.7% (5/711)	1.1% (5/442)	0% (0/269)	0.1628
Parasitemia (parasites/μL)	59 (5-551)	158 (17-900)	9 (3-118)	<0.0001
New visit 2 seroconversion	29.7% (211/711)	31.9% (141/442)	26.0% (70/269)	0.5418
New visit 3 seroconversion	21.8% (132/605)	22.7% (83/366)	17.8% (48/269)	0.1646
Visit 2 seropositives reverting to seronegative by visit 3	36.2% (64/177)	40.2% (43/107)	30.0% (21/70)	0.2013

Comorbidity refers to any of the following self-reported chronic conditions at the time of enrolment: obesity, diabetes, HIV or other immunosuppression, hypertension, cardiovascular disease, chronic lung disease, chronic hematological disorder, chronic kidney disease, chronic neurological impairment, malignancy.

P values denote pairwise comparison between clinical malaria and asymptomatic malaria groups.

Supplementary Table 3: Adult participants (≥18 years) and child participants (<18 years) with positive malaria diagnostic test during 2020 malaria transmission season stratified into clinical malaria and asymptomatic malaria groups

	Adults (n=326)			Children (n=385)		
	Clinical malaria (n=224)	Asymptomatic malaria (n=102)	P value	Clinical malaria (n=218)	Asymptomatic malaria (n=167)	P value
Age, years (median, IQR)	32.5 (25-45)	35 (25-43)	0.8731	11 (9-13)	6 (4-13)	<0.0001
Sex, male	39.3% (88/224)	48.0% (49/102)	0.1478	63.8% (139/218)	58.1% (97/167)	0.2570
Comorbidity	2.2% (5/224)	0% (0/102)	0.3299	0.5% (1/218	0% (0/167)	0.3808
Parasitemia (parasites/µL)	125 (16-1108)	7 (2-45)	<0.0001	189 (19-881)	13 (3-250)	<0.0001
New visit 2 seroconversion	41.5% (93/224)	35.3% (36/102)	0.3289	22.0% (48/218)	20.4% (34/167)	0.6935
New visit 3 seroconversion	37.1% (36/97)	38.5% (20/52)	>0.9999	29.0% (47/162)	23.1% (30/130)	0.2860
Visit 2 seropositives reverting to seronegative by visit 3	43.6% (27/62)	27.8% (10/36)	0.1359	35.6% (16/45)	32.4% (11/34)	0.8143

Supplementary Table 4: Symptom reporting rates in participants with new COVID-19 seroconversion during the 2020 malaria transmission season stratified by presence of parasitemia during the 2020 malaria transmission season

	COVID-19 seroconversion and parasitemia (n=211)	COVID-19 seroconversion only (no parasitemia) (n=181)	p-value
Any symptoms	56.9% (120/211)	56.9% (103/181)	>0.9999
Any systemic symptoms	27.01% (57/211)	33.15% (60/181)	0.9894
Any respiratory symptoms	42.18% (89/211)	42.54% (77/181)	>0.9999
Any gastrointestinal symptoms	5.21% (11/211)	3.87% (7/181)	>0.9999
Fever	5.69% (12/211)	4.97% (9/181)	>0.9999
Chills	0.5% (1/211)	1.7% (3/181)	0.9991
Fatigue	4.7% (10/211)	4.4% (8/181)	>0.9999
Muscle ache	1.9% (4/211)	1.7% (3/181)	>0.9999
Headache	24.17% (51/211)	30.39% (55/181)	0.9730
Sore throat	2.8% (6/211)	1.7% (3/181)	>0.9999
Cough	29.4% (62/211)	29.3% (53/181)	>0.9999
Runny nose	35.6% (75/211)	37.6% (68/181)	>0.9999
Shortness of breath	0.5% (1/211)	0% (0/181)	>0.9999
Wheezing	0% (0/211)	0% (0/181)	>0.9999
Loss of smell/taste	1.0% (2/211)	1.1% (2/181)	>0.9999
Other respiratory symptoms	0% (0/211)	0.6% (1/181)	>0.9999
Nausea/vomiting	2.4% (5/211)	2.2% (4/181)	>0.9999
Abdominal pain	2.8% (6/211)	2.2% (4/181)	>0.9999
Diarrhea	1.4% (3/211)	0.6% (1/181)	>0.9999

P values denote pairwise comparison between COVID-19 seroconversion and parasitemia group and COVID-19 seroconversion only group, corrected using Holm-Sidak method for multiple comparisons.

Reported symptoms were also compared between participants with new COVID-19 seroconversion and recent clinical malaria, and new COVID-19 seroconversion alone. Participants with asymptomatic malaria were not include. The proportion of asymptomatic COVID-19 seroconversions was the same between these groups (clinical malaria: 43.3% (61/141) vs. no parasitemia: 43.1% (78/181), p>0.9999). The number of individual symptoms reported in symptomatic participants and overall reported duration were also the same (clinical malaria: median number of symptoms 2 IQR 1 to 2 vs. no parasitemia: 2 IQR 1 to 3, p=0.6090; clinical malaria: median duration 4 days IQR 2 to 5 vs. no parasitemia: 4 days IQR 2 to 5, p=0.8400). There was no difference in the reporting rate of any individual symptom.

Supplementary Table 5: Symptom reporting rates in adult participants (≥18 years) and child participants (<18 years) with new COVID-19 seroconversion during the 2020 malaria transmission season stratified by presence of parasitemia during the 2020 malaria transmission season

	Adults (n=279)			Children (n=113)		
	COVID-19 seroconversion and parasitemia (n=129)	COVID-19 seroconversion only (no parasitemia) (n=150)	p-value	COVID-19 seroconversion and parasitemia (n=82)	COVID-19 seroconversion only (no parasitemia) (n=31)	p-value
Any symptoms	51.9% (67/129)	56.0% (84/150)	0.9998	64.6% (53/82)	61.3% (19/31)	>0.9999
Any systemic symptoms	28.7% (37/129)	34.7% (52/150)	0.9957	24.4% (20/82)	25.8% (8/31)	>0.9999
Any respiratory symptoms	31.8% (41/129)	40.0% (60/150)	0.9654	58.5% (48/82)	54.8% (17/31)	>0.9999
Any gastrointestinal symptoms	7.0% (9/129)	3.3% (5/150)	0.9663	2.4% (2/82)	6.5% (2/31)	0.9989
Fever	5.4% (7/129)	5.3% (8/150)	>0.9999	6.1% (5/82)	3.2% (1/31)	>0.9999
Chills	0% (0/129)	2.0% (3/150)	0.9903	1.2% (1/82)	0% (0/31)	>0.9999
Fatigue	6.2% (8/129)	5.3% (8/150)	>0.9999	2.4% (2/82)	0% (0/31)	>0.9999
Muscle ache	3.1% (4/129)	2.0% (3/150)	>0.9999	0% (0/82)	0% (0/31)	>0.9999
Headache	26.4% (34/129)	32.0% (48/150)	0.9969	20.7% (17/82)	22.6% (7/31)	>0.9999
Sore throat	0.8% (1/129)	0.7% (1/150)	>0.9999	6.1% (5/82)	6.5% (2/31)	>0.9999
Cough	23.3% (30/129)	26.7% (40/150)	0.9998	39.0% (32/82)	41.9% (13/31)	>0.9999
Runny nose	24.8% (32/129)	35.3% (53/150)	0.7366	52.4% (43/82)	48.4% (15/31)	>0.9999
Shortness of breath	0% (0/129)	0% (0/150)	>0.9999	1.2% (1/82)	0% (0/31)	>0.9999
Wheezing	0% (0/129)	0% (0/150)	>0.9999	0% (0/82)	0% (0/31)	>0.9999
Loss of smell/taste	1.6% (2/129)	1.3% (2/150)	>0.9999	0% (0/82)	0% (0/31)	>0.9999
Other respiratory symptoms	0% (0/129)	0.7% (1/150)	>0.9999	0% (0/82)	0% (0/31)	>0.9999
Nausea/vomiting	3.1% (4/129)	2.7% (4/150)	>0.9999	1.2% (1/82)	0% (0/31)	>0.9999
Abdominal pain	3.1% (4/129)	1.3% (2/150)	0.9986	2.4% (2/82)	6.5% (2/31)	0.9989
Diarrhea	2.3% (3/129)	0.7% (1/150)	0.9969	0% (0/82)	0% (0/31)	>0.9999

P values denote pairwise comparison between COVID-19 seroconversion and parasitemia group and COVID-19 seroconversion only group, corrected using Holm-Sidak method for multiple comparisons.

Supplementary Table 6: Symptom reporting rates in participants with new COVID-19 seroconversion in the post-transmission dry season stratified by presence of parasitemia during the 2020 malaria transmission season

	COVID-19 seroconversion and prior parasitemia (n=133)	COVID-19 seroconversion only (no parasitemia) (n=108)	p-value
Any symptoms	25.6% (34/133)	32.4% (35/108)	0.9845
Any systemic symptoms	11.3% (15/133)	15.7% (17/108)	0.9920
Any respiratory symptoms	22.6% (30/133)	26.9% (29/108)	0.9934
Any gastrointestinal symptoms	5.3% (7/133)	3.7% (4/108)	0.9934
Fever	2.3% (3/133)	3.7% (4/108)	0.9934
Chills	2.3% (3/133)	0.9% (1/108)	0.9934
Fatigue	2.3% (3/133)	1.9% (2/108)	0.9991
Muscle ache	0.8% (1/133)	2.8% (3/108)	0.9816
Headache	11.3% (15/133)	13.9% (15/108)	0.9934
Sore throat	3.8% (5/133)	1.9% (2/108)	0.9934
Cough	18.8% (25/133)	24.1% (26/108)	0.9920
Runny nose	17.3% (23/133)	25.0% (27/108)	0.9369
Shortness of breath	0% (0/133)	0% (0/108)	>0.9999
Wheezing	0% (0/133)	0% (0/108)	>0.9999
Loss of smell/taste	0.8% (1/133)	0% (0/108)	0.9934
Other respiratory symptoms	0% (0/133)	0% (0/108)	>0.9999
Nausea/vomiting	1.5% (2/133)	3.7% (4/108)	0.9891
Abdominal pain	4.5% (6/133)	0.9% (1/108)	0.8629
Diarrhea	1.5% (2/133)	0% (0/108)	0.9778

P values denote pairwise comparison between COVID-19 seroconversion and parasitemia group and COVID-19 seroconversion only group, corrected using Holm-Sidak method for multiple comparisons.

Supplementary Table 7: Symptom reporting rates in adult participants (≥18 years) and child participants (<18 years) with new COVID-19 seroconversion in the post-transmission dry season stratified by presence of parasitemia during the 2020 malaria transmission season

	Adults (n=117)			Children (n=124)		
	COVID-19 seroconversion and prior parasitemia (n=56)	COVID-19 seroconversion only (no parasitemia) (n=61)	p-value	COVID-19 seroconversion and prior parasitemia (n=77)	COVID-19 seroconversion only (no parasitemia) (n=47)	p-value
Any symptoms	37.5% (21/56)	36.1% (22/61)	>0.9999	16.9% (13/77)	27.7% (13/47)	0.9167
Any systemic symptoms	21.4% (12/56)	24.6% (15/61)	>0.9999	3.9% (3/77)	4.3% (2/47)	>0.9999
Any respiratory symptoms	33.9% (19/56)	27.9% (17/61)	>0.9999	14.3% (11/77)	25.5% (12/47)	0.8817
Any gastrointestinal symptoms	8.9% (5/56)	3.3% (2/61)	0.9936	2.6% (2/77)	4.3% (2/47)	>0.9999
Fever	5.4% (3/56)	6.6% (4/61)	>0.9999	0% (0/77)	0% (0/47)	>0.9999
Chills	5.4% (3/56)	1.6% (1/61)	0.9989	0% (0/77)	0% (0/47)	>0.9999
Fatigue	5.4% (3/56)	3.3% (2/61)	>0.9999	0% (0/77)	0% (0/47)	>0.9999
Muscle ache	1.8% (1/56)	4.9% (3/61)	>0.9999	0% (0/77)	0% (0/47)	>0.9999
Headache	21.4% (12/56)	21.3% (13/61)	>0.9999	3.9% (3/77)	4.3% (2/47)	>0.9999
Sore throat	5.4% (3/56)	1.6% (1/61)	0.9989	2.6% (2/77)	2.1% (1/47)	>0.9999
Cough	26.8% (15/56)	24.6% (15/61)	>0.9999	13% (10/77)	23.4% (11/47)	0.8990
Runny nose	28.6% (16/56)	26.2% (16/61)	>0.9999	9.1% (7/77)	23.4% (11/47)	0.4193
Shortness of breath	0% (0/56)	0% (0/61)	>0.9999	0% (0/77)	0% (0/47)	>0.9999
Wheezing	0% (0/56)	0% (0/61)	>0.9999	0% (0/77)	0% (0/47)	>0.9999
Loss of smell/taste	1.8% (1/56)	0% (0/61)	>0.9999	0% (0/77)	0% (0/47)	>0.9999
Other respiratory symptoms	0% (0/56)	0% (0/61)	>0.9999	0% (0/77)	0% (0/47)	>0.9999
Nausea/vomiting	3.6% (2/56)	3.3% (2/61)	>0.9999	0% (0/77)	4.3% (2/47)	0.7185
Abdominal pain	7.1% (4/56)	1.6% (1/61)	0.9826	2.6% (2/77)	0% (0/47)	0.9866
Diarrhea	3.6% (2/56)	0% (0/61)	0.9903	0% (0/77)	0% (0/47)	>0.9999

P values denote pairwise comparison between COVID-19 seroconversion and parasitemia group and COVID-19 seroconversion only group, corrected using Holm-Sidak method for multiple comparisons.

Supplementary Figure 1: Violin plots of SARS-CoV-2 spike and RBD signal rate change (OD units/100 days) during 2020 malaria transmission season stratified by presence of parasitemia during season for A) all participants (n=1314), B) visit 2 seronegative participants (n=922), C) visit 2 seropositive participants (n=392)

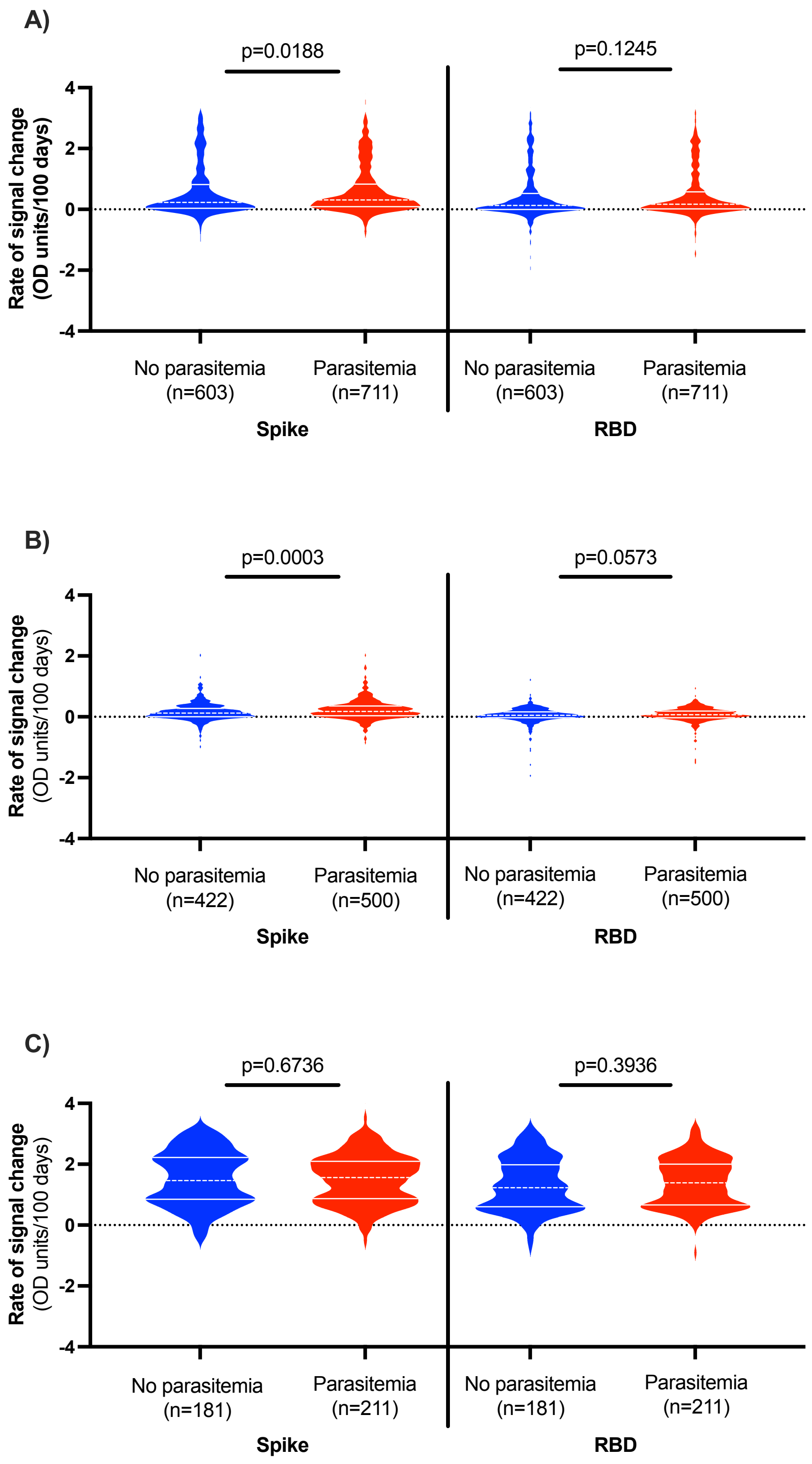

Supplementary Figure 2: Violin plots of SARS-CoV-2 spike and RBD signal rate change (OD units/100 days) over the 2020 malaria transmission season (between study visit 1 and 2) stratified by presence of parasitemia during the season for A) all adult participants (n=701), B) visit 2 seronegative adults (n=423), C) visit 2 seropositive adults (n=279), D) child participants (n=613), E) visit 2 seronegative children (n=500), F) visit 2 seropositive children (n=113)

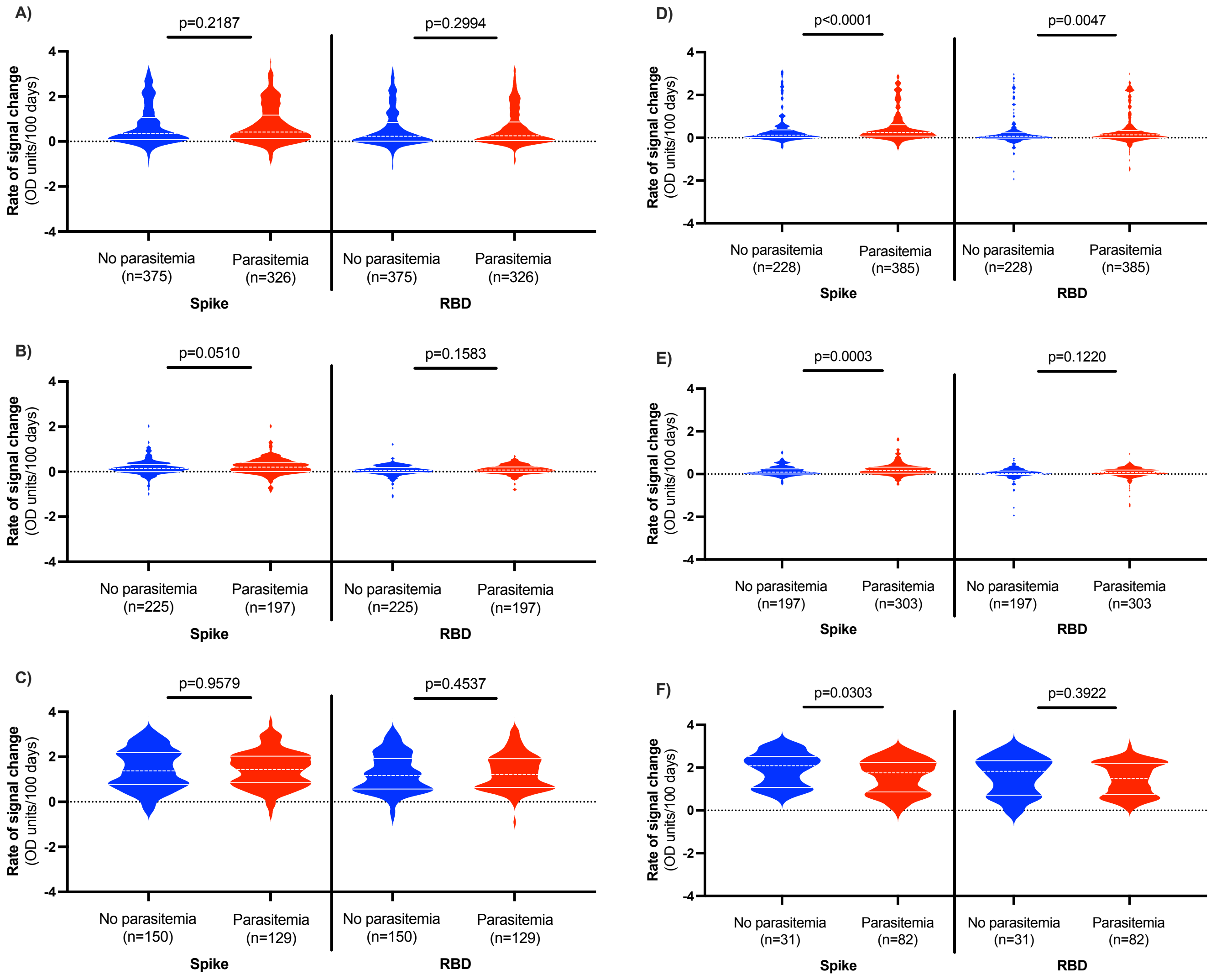

Supplementary Figure 3: Association of covariates with clinical malaria during 2020 malaria transmission season by logistic regression (n=1314, forest plot of odds ratios and 95% confidence intervals)

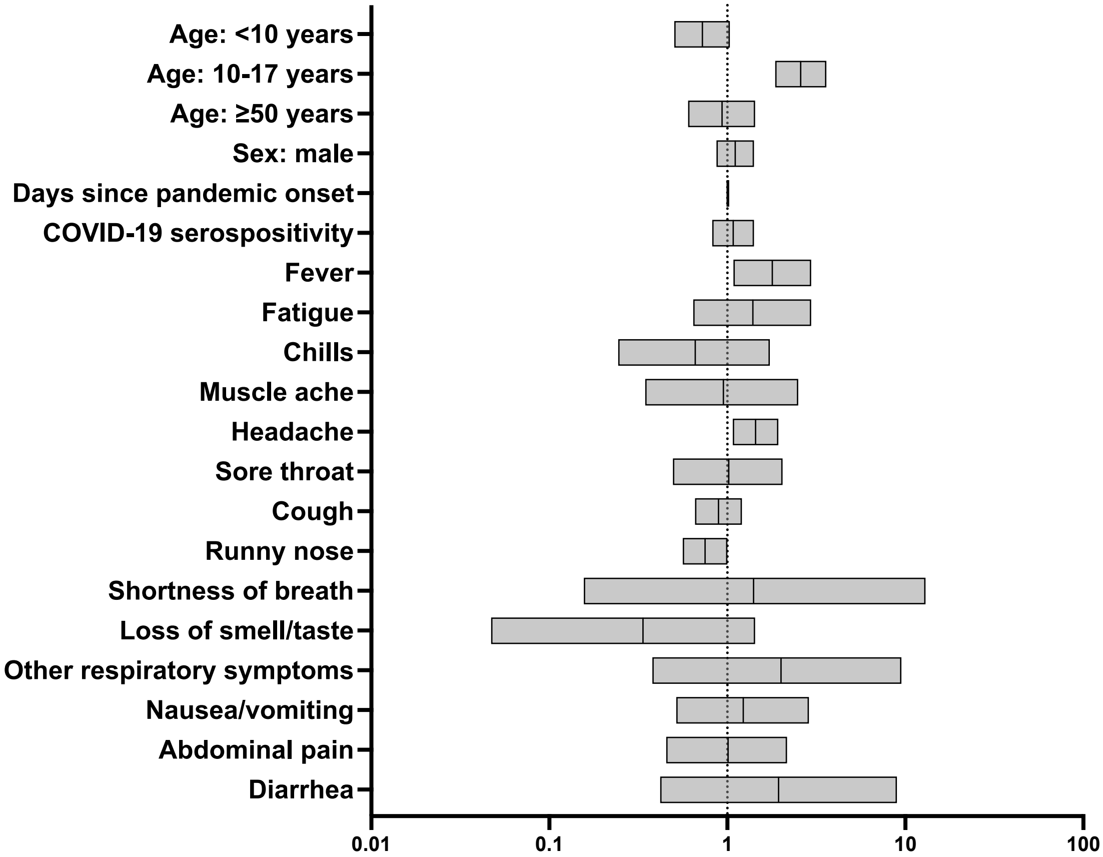

Age group 18-49 years and Doneguebougou site used as reference groups. Wheezing not included in analysis due to lack of reported events.

Supplementary Figure 4: Violin plots of SARS-CoV-2 spike and RBD signal rate change (OD units/100 days) during the post-transmission dry season for participants seropositive at the end of the transmission season, stratified by recent prior parasitemia A) participants that remain seropositive at visit 3 (n=195), B) participants that revert to seronegative at visit 3 (n=101)

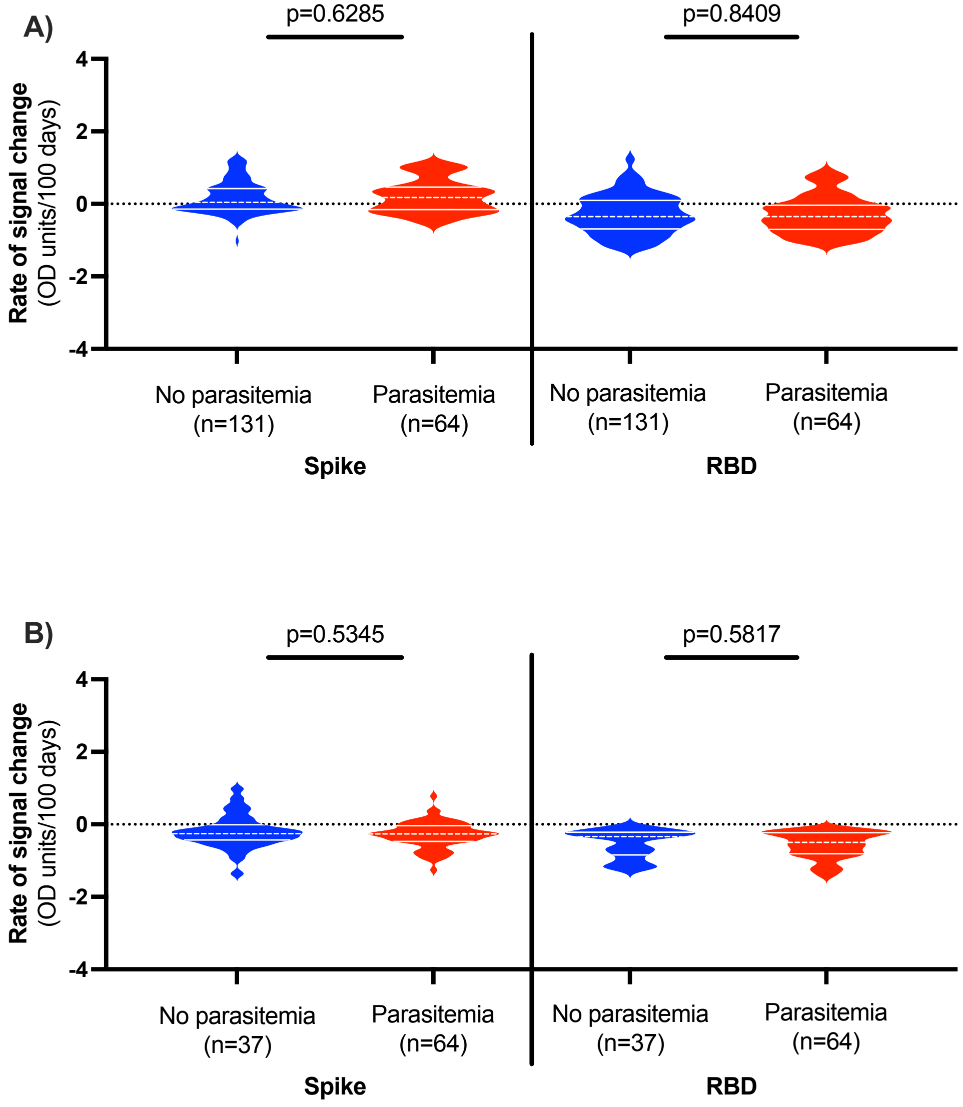

Supplementary Figure 5: Violin plots of SARS-CoV-2 spike and RBD signal rate change (OD units/100 days) during the post-transmission dry season for participants seropositive study visit 2, stratified by recent prior parasitemia A) adults that remained seropositive at visit 3 (n=123), B) adults that reverted to seronegative at visit 3 (n=65), C) children that remained seropositive at visit 3 (n=72), D) children that reverted to seronegative at visit 3 (n=36)

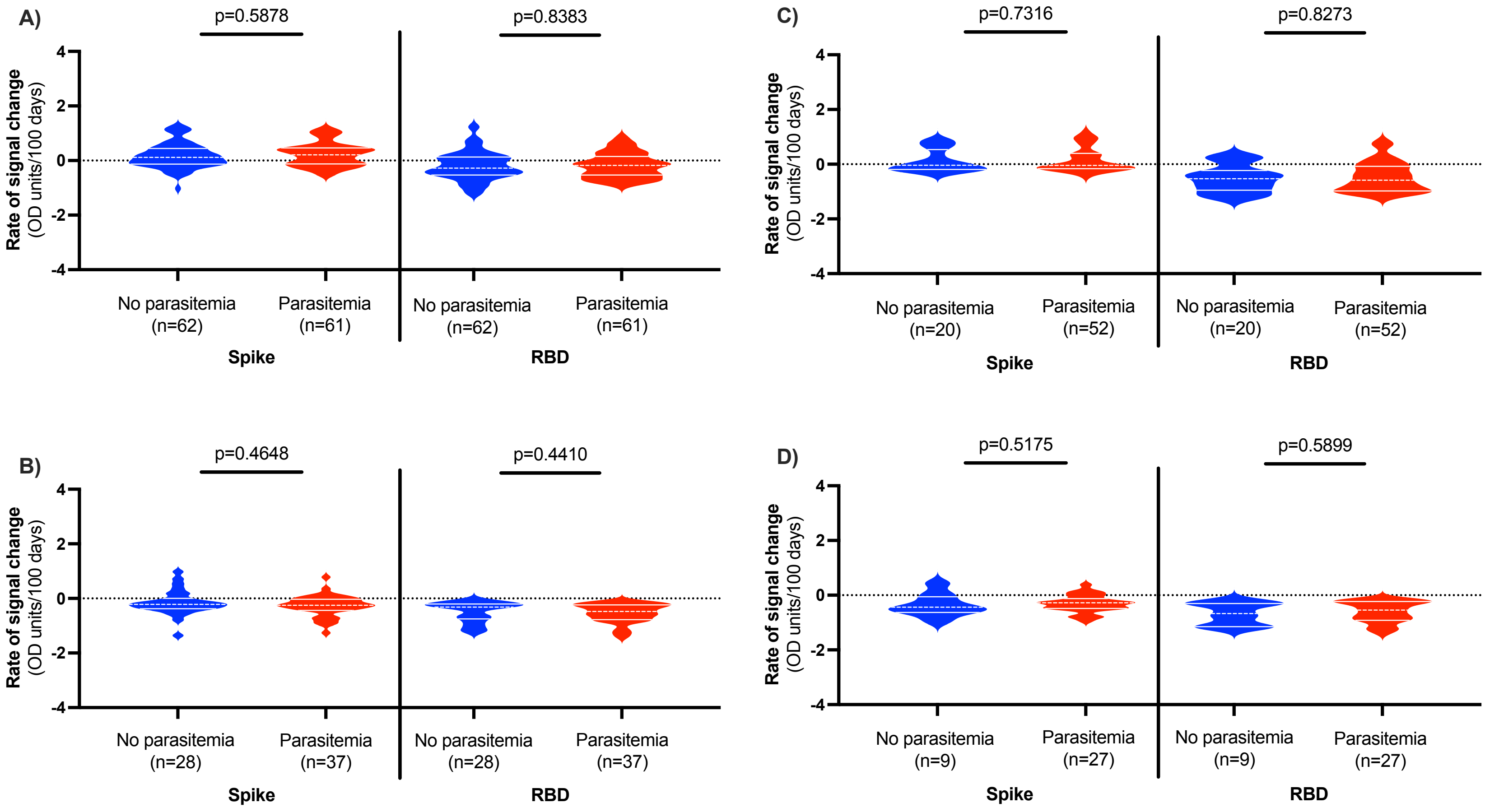

Supplement: Supplementary file 1 [file DataSheet_1.pdf]
